# Application of multidisciplinary team conference for neuromodulation candidates facilitates patient selection and optimization

**DOI:** 10.3389/fpain.2023.1331883

**Published:** 2024-01-05

**Authors:** Vafi Salmasi, Mohammad Reza Rasouli, Ming C. Kao, Einar Ottestad, Abdullah Sulieman Terkawi, Garret Morris, Xiang Qian, Stephen Coleman, David C. Talavera, Heather Poupore-King, Kristen Slater, Michael S. Leong

**Affiliations:** Department of Anesthesiology, Perioperative and Pain Medicine, Division of Pain Medicine, Stanford University School of Medicine, Palo Alto, CA, United States

**Keywords:** neuromodulation, multidisciplinary, spinal cord stimulation, psychological evaluation, team conference

## Abstract

**Introduction:**

Psychological evaluation is required by insurance companies in the United States prior to proceeding with a spinal cord stimulation or a dorsal root ganglion stimulation trial. Since January 2017, we implemented a Multidisciplinary Team Conference for Neuromodulation in our center to facilitate the collaboration between pain physicians and psychologists and to optimize screening of neuromodulation candidates. This study aims to report the impact of this team conference on improvement of neuromodulation outcome in our center.

**Methods:**

Appropriateness of neuromodulation were discussed in the team conference after initial visit with the pain specialist and psychological evaluation. For this study, we prospectively and retrospectively collected data on neuromodulation candidates who went through the team conference and those who did not as controls.

**Results:**

We discussed 461 patients in the team conference sessions from January 2017 to July 2023. Out of these, a spinal cord stimulator or a dorsal root ganglion stimulator trial was performed in 164 patients with 80.5% (132 cases) trial success rate leading to 140 implants. Out of these implants, 26 (18.6%) explanted and 21 (15%) required revision in 41 (29.3%) patients. We performed neuraxial neuromodulation trial for 70 patients without going through the team conference from January 2016 to July 2023 with a trial success rate of 45.7% (32 cases). In this group, 7 (21.9%) and 6 (18.8%) patients underwent explant and revision. The differences between the groups were statistically significant for trial success rate (odds ratio of 4.9 with *p*-value of <0.01) but not for explant (odds ratio of 0.8 with *p*-value of 0.627) or revision (odds ratio of 0.8 with *p*-value of 0.595).

**Conclusion:**

Implementing Multidisciplinary Team Conference increased trial success rate in our center. Team conference provides therapeutic benefit for patients, and also provides the opportunity for an educational discussion for trainees.

## Introduction

Neuromodulation is an effective treatment for patients with chronic pain. Several studies have demonstrated the efficacy of spinal cord stimulation in reducing pain and improving quality of life in patients with chronic pain; thus, successful implementation of neuromodulation as a component of multidisciplinary treatment plan can decrease the cost of care for patients with chronic pain (1–4). However, the high initial cost associated with trials and implantation of neurostimulators highlight the importance of better patient selection and patient optimization (medical and psychological). The Neuromodulation Appropriateness Consensus Committee (NACC) has provided guidelines for the appropriate use of neurostimulation for chronic pain (5). These guidelines help in identifying patients who are most likely to benefit from spinal cord stimulation, ensuring that the treatment is cost-effective. Psychological (narrative) evaluation report is required by insurance companies in the United States prior to proceeding with a trial of spinal cord stimulation. This evaluation aims to assess the psychological factors that may influence the efficacy of neuromodulation and ensure that patients are suitable candidates for the procedure. The evaluation process should include strict inclusion and exclusion criteria, pre-operative psychosocial assessment, and consistent psychological and rehabilitative support throughout the trial phase and subsequent therapy (6).

After assuring appropriate patient selection, patient optimization is an important factor in improving outcomes of surgical interventions. American College of Surgeons recommends focusing on at least four optimization categories prior to surgery: nutrition (lab tests like serum albumin for risk stratification and malnutrition screening, and inquiring about use of supplements), blood sugar (optimizing long-term blood sugar control monitored by measurement of hemoglobin A1C), smoking (quitting smoking at least 4–6 weeks before surgery to normalize immune and metabolic function) and medications (thorough review of all medications taken by patients (including anti-coagulants), and making recommendations (through mutual decision making with other specialties involved) about safety of stopping or continuing medications perioperatively). These recommendations align with the principles of enhanced recovery after surgery and aim to improve patient outcomes and enhance recovery. Following these guidelines can contribute to better surgical outcomes and patient satisfaction (7, 8). Chronic pain is a biopsychosocial disease (9, 10); thus, it is important to optimize patients psychologically in addition to above-mentioned steps to medically optimize them. Psychological optimization is important to assure: (1) the proposed surgical interventions aligns with other aspects of patients' multidisciplinary treatment plan; (2) the patient is psychologically stable with no untreated psychological disease; and (3) the patient is capable of applying appropriate coping strategies if the neuromodulation is not successful.

In order to facilitate the collaboration between pain physicians and pain psychologists in this patient selection and optimization process, some academic institutions have implemented additional approaches to improve the success rate of neuromodulation (e.g., Stanford University, Cleveland Clinic, and University of California Davis). One of these approaches is use of multidisciplinary team conferences that specifically focus on implant candidacy and optimization. We are reporting the implementation of this process at the Stanford Pain Management Center and how it has improved neuromodulation outcome in our center.

## Methods

### Neuromodulation team conference process

Patients considered for neuromodulation are added to the department shared list of neuromodulation candidates. After the initial visit with a pain medicine specialist, patients consult with a pain psychologist for a targeted evaluation. The evaluation typically involves either an in-person or online interview with the patient, as well as the use of self-reporting assessment tools. The clinical discussion delves into various psychological factors known to affect pain, mood, functionality, and treatment results. These factors may include past traumas, mental health status, substance habits, current stressors, overall past and present functioning, the effect of pain on their life, and their social support network. Key to the neuromodulation evaluation are extra questions to gauge the patient's understanding of the procedure, expectations, ability to deal with mixed or negative results, and history of following treatment guidelines. Self-administered psychological tests can offer more targeted data, helping to form a well-rounded view of the patient's suitability for such treatment options. After the assessment, a detailed report is produced that combines findings from both the self-report tools and the clinical interview. The psychologist will pinpoint any potential risks that could affect neuromodulation outcomes in the report's final impressions and suggestions. This report doesn't necessarily give a final verdict on whether the patient should undergo the procedure or not. Instead, it outlines any red flags and their potential implications, often sharing these insights with the whole committee to help refine treatment plans.

After completion of these two visits, the patient cases are discussed during neuromodulation multidisciplinary team conference sessions. These sessions take place once or twice a month; pain medicine faculty, pain medicine fellows, pain psychologists and pain psychology fellows attend these conferences. First, the pain medicine faculty member or fellow responsible for care of a patient presents the patient followed by pain psychology fellow or faculty member. We discuss the following:

1. Pain history
2. Appropriate diagnosis and indication for neuromodulation
3. Previous pain treatments
4. Medical comorbidities and medications
5. Imaging and lab tests
6. Psychological comorbidities
7. Substance use, alcohol use and smoking
8. Overall multidisciplinary treatment plan
9. Patient goals

Based on these factors, the team decides upon the following:

1. Is the patient a good candidate for neuromodulation?
2. What steps are necessary to medically optimize the patient?
3. What steps are necessary to psychologically optimize the patient?
4. What is the best neuromodulation modality for the patient? These modalities include peripheral nerve stimulation (temporary or permanent), dorsal root ganglion stimulation, spinal cord stimulation (and the specific manufacturer based on available features and waveforms in concordance with patient diagnosis, abilities and goals), transcranial magnetic stimulation, etc.

**Figure 1:**
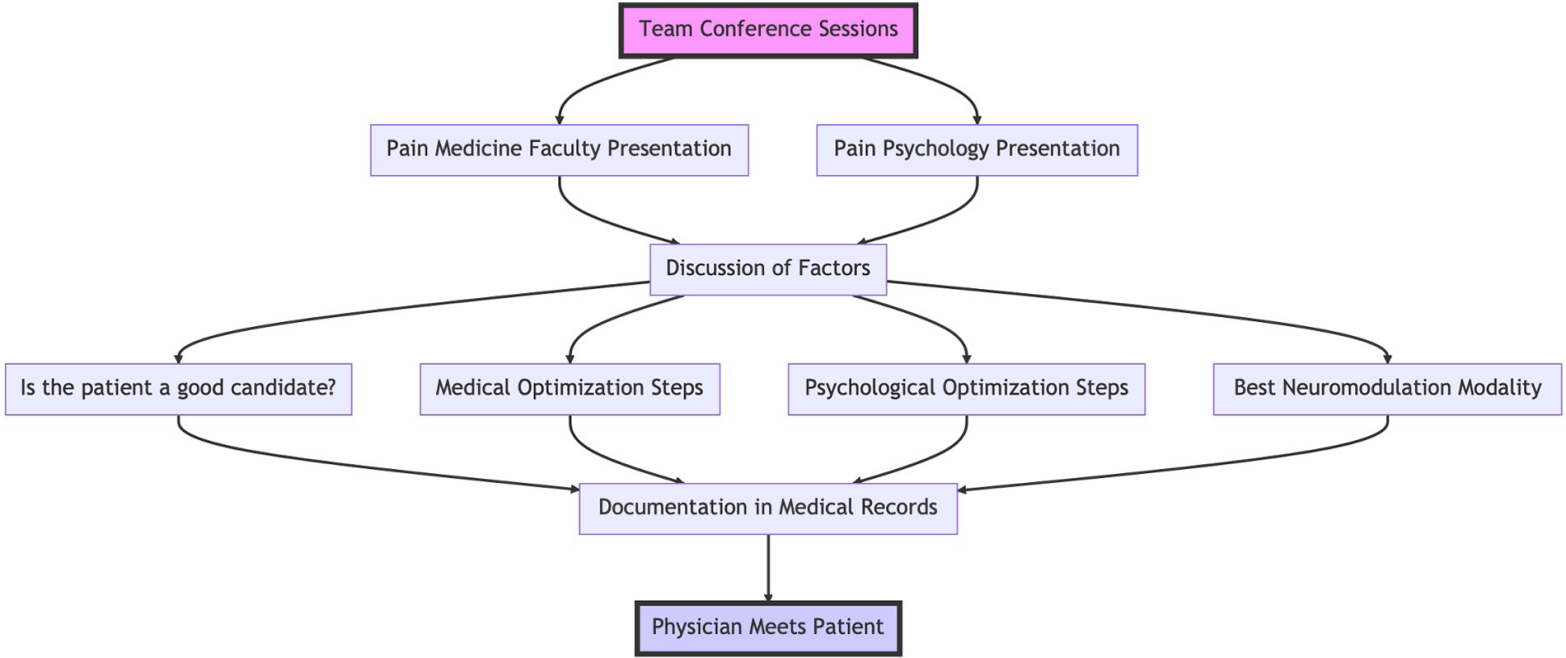
Summary of the team conference process.

The response to these questions develops the multidisciplinary treatment plan focused on neuromodulation. The discussions are then documented in patients' medical records. The physician responsible for patients' care will then meet with them to discuss and implement the recommendations of the team conference. Figure 1 summarizes the team conference process.

### Data collection

We received approval from Stanford University Institutional Review Board to prospectively collect data on all patients who go through screening for neurostimulation in our clinic. Since the beginning of the process, we have been prospectively collecting data on all patients considered for neuromodulation. We collect data about patient's age, sex, dates of last clinical and psychology visits, date of team conference, date of neuromodulation trial or implant, success of the trial (defined as patients' subjective reported improvement of more than 50% in either pain intensity or physical function) recommendations from team conference, type of neurostimulation device recommended by the team conference, type of neurostimulation device used for trial and implant, possible revision and explant of the device. We collected similar data both prospectively and retrospectively for any patients who went through process of spinal cord stimulator trial without being screened by the team conference during the same period and one year before that as controls. Participation in the team conference for screening is strongly recommended within our group, but it is not a mandatory prerequisite for proceeding with neuromodulation. As a result, some clinicians have opted to bypass this collaborative step. Our initiative for neuromodulation team conferences kicked off in early 2017. To ensure a fair comparison of outcomes, we deliberately chose not to include data more than a year prior to the initiation of these conferences for controls. This decision was made to minimize variability and ensure that we were evaluating comparable technological approaches. We compared these outcomes using Fisher's Exact Test. We decided to use a non-parametric test considering that: (1) there were a couple of cells with lower frequency (6 and 7); and (2) the sample size in both groups were vastly different.

## Results

**Figure 2:**
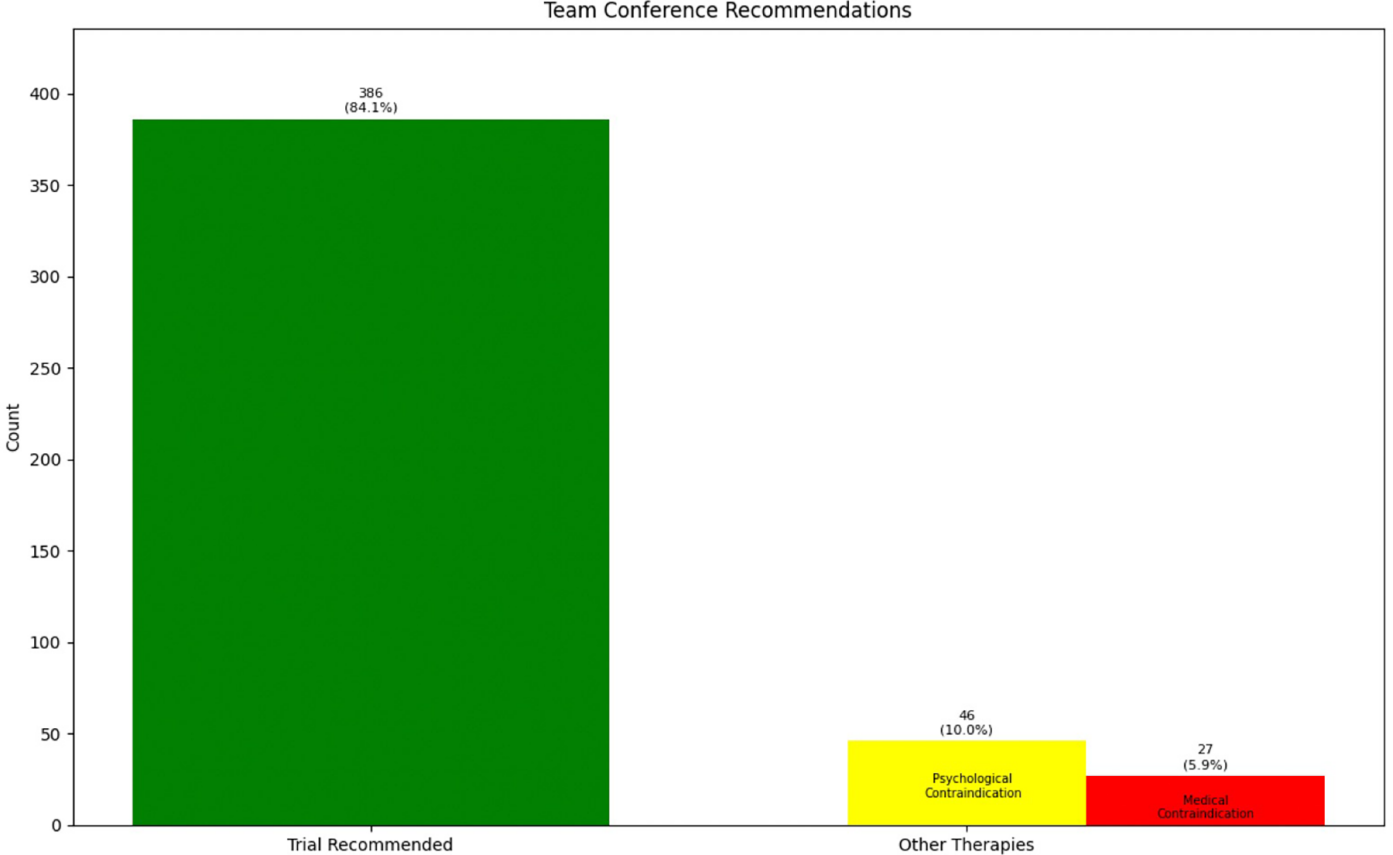
Initial therapy recommended after team conference.

**Figure 3:**
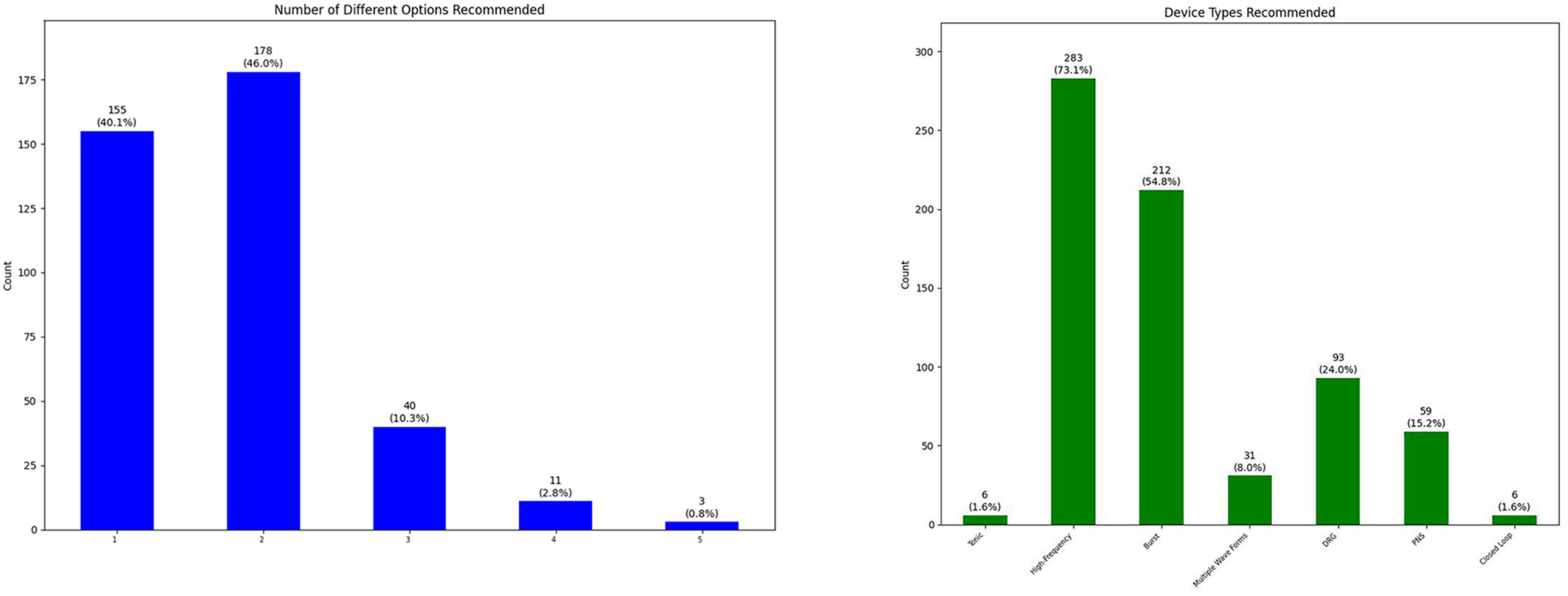
Number of different neuromodulation options recommended for each patient after team conference (left), and breakdown of type of neuromodulation devices recommended for patients after team conference (right) (DRG, dorsal root ganglion stimulation; PNS, peripheral nerve stimulation).

We have discussed the appropriateness of neuromodulation for 461 patients. There were 259 (56.2%) male patients and 202 (43.8%) female patients in our cohort with average age of 59.1 years (ranging from 18 to 92 years old with standard deviation of 15.5 years). In some occasions our physicians decided to bypass the process of team conference because of multiple reasons [not being familiar with the process (when we first started adding this step), thinking the patient is “straight-forward” and ready for the procedure, thinking the team conference process might delay the patients' trial, etc.]. Our team decided that 73 patients (15.9%) were inappropriate candidates for neuromodulation because of medical comorbidities, anatomical/psychological contraindications or better indication for other treatments: intrathecal pump, behavioral techniques, surgical interventions, etc. The other 386 (84.1%) patients were considered appropriate candidates (Figure 2). Our team recommendation included options for more than one type of neuromodulation device for more than half of the patients (Figure 3). Figure 3 also shows the breakdown of different types of technology recommended during these sessions.

**Figure 4:**
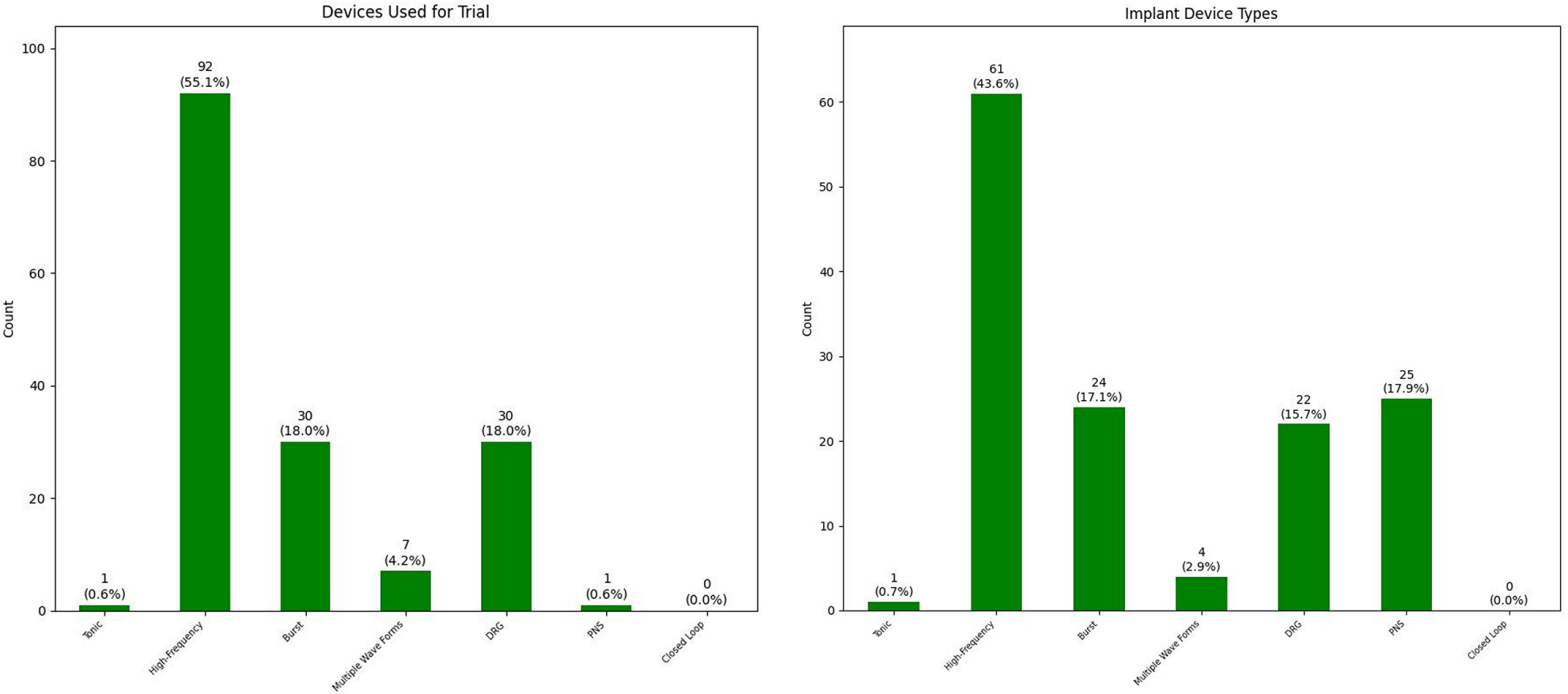
Breakdown of type of neuromodulation devices used for trial (left) and implant (right) (DRG, dorsal root ganglion stimulation; PNS, peripheral nerve stimulation).

A trial of neuraxial neurostimulation device (spinal cord or dorsal root ganglion stimulator) has been performed in 164 patients with 132 successful trials (80.5% trial success). We performed a peripheral nerve stimulator trial for one patient which was successful. For 30 (7.8%) patients, we directly proceeded with implant of a peripheral nerve stimulator device. The remaining 191 patients are either not interested, went to other practices because of longer wait times at Stanford, or currently undergoing optimization steps recommended during team conference. Figure 4 summarizes the types of neurostimulation technology used for trials.

The average length of trial was 7 days with standard deviation of 1.4. The majority of trials were 5–10 days long with four exceptions:

1. Two trials that prolonged to 12 and 14 days to allow for change to a second system in the middle of trial period. None of these trials were successful.
2. Two trials that prematurely ended at postoperative day number one. One patient had coincidental acute pancreatitis attack; we removed the leads to make sure we give the patient the opportunity for a trial at more optimized conditions in future. One patient had intrathecal placement of leads and we had to remove the leads after confirming the intrathecal placement by CT scan.

We have done 140 implants in this group of patients; we did not do a trial for all patients who received a peripheral nerve stimulator. Figure 4 also summarizes the types of neurostimulation technology used for implants. The average time between successful trial and implant was 59.2 days with standard deviation of 32.8 days. We observed a few patients with remarkably prolonged time intervals between their successful trial and implant: (1) two cases who had their successful trial right before COVID-19 pandemic and their implants were postponed because of that (196 days and 234 days); (2) one case whose insurance denied the implant after successful dorsal root ganglion stimulator implant for pelvic pain (approved after second appeal; 203 days); (3) one case whose first attempt at implant was aborted because of vomiting on operating room table before the beginning of the procedure (286 days after full medical evaluation and optimization); and (4) one case who had other life events in their family and had to postpone the implant for more than a year (384 days).

**Figure 5:**
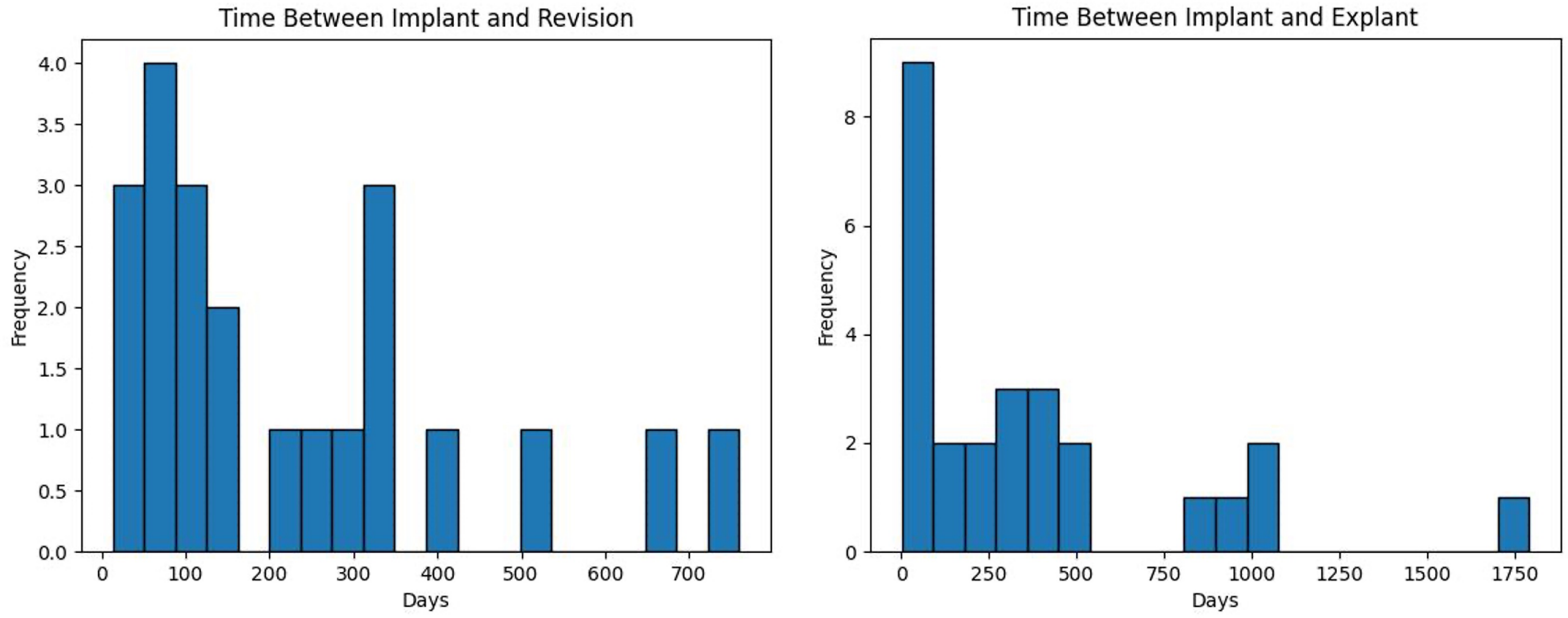
The distribution of time between implant and revision (left) or explant (right) of a neuromodulation device in days.

We performed 26 (18.6%) explants and 21 (15%) revisions in 41 (29.3%) patients. There were 6 patients who initially had a revision but later we had to explant the device. Average time between implant and explant was 371.2 days (1–1,792 days with standard deviation of 430.7 days). Average time between implant and revision was 231.8 days (13–760 days with standard deviation of 206.7 days). These intervals are not normally distributed with majority of explants and revisions occurring within the first year after implant (Figure 5).

The most common reason for explant was loss of efficacy despite multiple sessions of programming in 11 (7.9%) patients followed by infection in 6 (4.2%) patients. Two patients requested explant because they needed an MRI. Two other patients requested explant since their pain significantly improved and they did not need the device anymore. The other reasons for explant (one patient each) were pain at battery pocket, pain at anchors, neurological deficit on first postoperative day, and epidural hematoma. In an unusual incident, we were attempting to revise a device secondary to lead migration when patient developed marked drop in oxygen saturation and we had to turn the patient back to supine position without being able to close the wounds. We covered the wounds with sterile coverage before turning the patient; nevertheless, we decided to explant the device to decrease risk of infection.

The most common reason for revision was lead migration in 11 (7.9%) patients followed by discomfort at battery pocket in 7 (5%) patients. We performed a revision surgery for three patients because of incomplete coverage of painful area during initial implant; we later explanted the devices for these three patients because of lack of efficacy.

During this period (starting January 2017 until July 2023) and one year before that (January 2016 to January 2017) our team performed trial of axial neurostimulation for 70 patients without going through the team conference. The success rate of trial was only 45.7% (32 successful trials) in these patients. Out of these 32 implants, seven patients (21.9%) underwent explant and 6 patients (18.8%) underwent revision. The comparison between these two groups is summarized in Table 1.

**Table 1:** Comparing outcomes for patients who did and did not go through screening process of our multidisciplinary team conference.

	With team conference	Without team conference	Odds ratio (95% confidence interval)	*p*-value
Trial success rate	132/164 (80.5%)	32/70 (45.7%)	4.9 (2.67–9)	<0.01
Explant rate	26/140 (18.6%)	7/32 (21.9%)	0.8 (0.32–2.09)	0.627
Revision rate	21/140 (15%)	6/32 (18.8%)	0.8 (0.28–2.08)	0.595

The results are based on Fisher's Exact Test.

## Discussion

We are reporting successful implementation of a multidisciplinary team conference in our center to better select and optimize patients for neuromodulation. Between January 2017 and July 2023, our team discussed appropriateness of 461 patients for neuromodulation. Approximately 15.9% of patients were deemed inappropriate for neuromodulation due to various factors such as medical comorbidities, anatomical or psychological contraindications, or a better fit for alternative treatments. This underscores the importance of a multidisciplinary approach in the selection process, balancing the medical, anatomical, and psychological factors to optimize patient outcomes.

During these sessions our team also focused on patient optimization: both medical and psychological. We believe close collaboration with our pain psychology colleagues was essential in success of this process since psychological factors are shown to be better predictors (compared with duration of pain, baseline disability, etc.) of success of spinal cord stimulation (6, 11). Our patient selection process increased the success rate of trial of neuraxial neurostimulation to more than 80% in our center. Our current success rate is slightly better than reports from big academic centers (68%–73%) in real world setting (12, 13) but still shy of reports from industry sponsored clinical trials (90% and more) in more controlled settings (14–17). Our current success rate through team conference is significantly higher compared to patients who did not go through team conference. However, this comparison is limited by: (1) the big difference in sample size of both groups. We decided not to include patients who went through trial of neuraxial neurostimulation more than one year before implementation of the team conference process to compare similar technology in both group which resulted in a much smaller sample size in the group who bypassed the team conference; (2) selection bias in these groups. The physicians in our clinic could make the decision to bypass the screening process by the conference. Bypassing this step usually happened either because the physicians were not familiar with this process at the beginning, or they were under time pressure by the patients; and (3) significantly lower success rate of trial of neuraxial neurostimulation in our control group (less than 50%) which is below the reports from any other academic center. Lower success rate can be a random event considering smaller size of control sample or secondary to selection bias by physicians who bypassed the team conference selection process.

We observed marginal improvement in incidences of revision and explant in patients who went through the team conference process. However, this improvement was neither clinically nor statistically significance. The small size of difference could be because of small sample size of the control group but we cannot make any conclusions based on the current findings. The complication rate in both groups is slightly lower but comparable to rates reported by Cleveland Clinic and Case Western (12, 13). We had to revise 21 (15%) devices and explant 26 (18.6%) devices in 41 (29.3%) patients; these rates were as high as 23.9% in the report by Hayek et al. (13). The incidence of infection (4.2%) in our cohort was similar to these reports (4.3% and 4.5%) (12, 13). Our lead migration rate (7.9%) was comparable to 8.5% reported by Hayek et al. (13) but much lower than 22.6% reported by Mekhail et al. (12). We believe that advances in anchoring technology is the main reason for lower rate of lead migration in more recent papers compared to Mekhail *et al*. (12). The incidence of discomfort at battery site leading to revision or explant was much lower in our cohort (5.7%) comparing to 11.1% reported by Hayek et al. (13).

There were a few noteworthy findings in our cohort: (1) we performed a revision surgery for 3 patients because of insufficient coverage of the painful area; however, all these revision surgeries were unsuccessful, and we had to explant these devices later; (2) we had to explant the devices in two patients who needed MRIs. This highlights how important it is for all neuromodulation devices to be MRI compatible; and (3) two patients requested to explant the device since their pain had been improved to the point that they did not need to use the device anymore. The main question is appropriateness of initial device implant for these patients. It is unclear if these patients' pain would have improved regardless of therapy. We were also debating if controlled pain for the short period these patients were using neurostimulators outweighed the potential risk of three procedures (trial, implant and explant).

## Limitations

Our study is not without limitations, including its retrospective nature and the absence of a control group of appropriate size. Moreover, there is selection bias in our control group; the choice of bypassing the team conference was definitely not random and did affect the outcomes of these patients.

We focused only on objective measures that can be easily captured through medical records; we thus miss important details about efficacy of the devices as well as patient burden and patient satisfaction. We are currently collecting relevant patient reported outcomes for all our neuromodulation patients prospectively for future studies.

## Conclusion

In larger academic institutions, addition of one last screening step prior to trial of a neuraxial or peripheral neuromodulation device is valuable. This step can identify appropriate patients for interventions and specify details necessary to optimize patients medically and psychologically prior to trial procedure. By implementing this optimization step, our trial success rate has increased to 80.5% which exceeds other large academic institutional rates (67%–73%). Moreover, comprehensive neuromodulation discussion can provide therapeutic benefit for patients, and also the opportunity for an educational discussion for trainees.

## Data Availability

The raw data supporting the conclusions of this article will be made available by the authors, without undue reservation.
